# Moderators’ Role in Fostering Health Practitioner Engagement Within an Online Active Learning Program (The Community Fracture Capture Learning Hub): Qualitative Relational Content Thematic Analysis

**DOI:** 10.2196/83764

**Published:** 2026-03-04

**Authors:** Ahmed M Fathalla, Shanton Chang, Ralph Audehm, Cherie Chiang, Alexandra Gorelik, Christopher J Yates, Steve Snow, Rahul D Barmanray, Sarah Price, Lucy Collins, John D Wark

**Affiliations:** 1 Department of Medicine, The Royal Melbourne Hospital The University of Melbourne Melbourne, Victoria Australia; 2 School of Computing and Information Systems University of Melbourne Melbourne Australia; 3 Department of General Practice and Primary Care University of Melbourne Melbourne Australia; 4 Department of Diabetes & Endocrinology, Royal Melbourne Hospital Melbourne Health Melbourne Australia; 5 School of Public Health and Preventive Medicine Monash University Melbourne Australia; 6 Praxhub Melbourne Australia; 7 Department of Obstetric Medicine, Royal Women's Hospital University of Melbourne Melbourne Australia; 8 Department of Medicine, School of Clinical Sciences Monash University Melbourne Australia

**Keywords:** community-based fracture capture learning hub, virtual community of practice, web-based learning moderator, primary care physicians, case-based learning, osteoporosis education, qualitative content analysis, continuing professional development, facilitation strategies, moderator-participant interaction, collaborative learning

## Abstract

**Background:**

Virtual communities of practice (VCoPs) offer flexible platforms for interprofessional education among primary care physicians (PCPs); yet, the role of moderators in optimizing engagement remains underexplored. The Community Fracture Capture Learning Hub, a case-based, interactive web-based program addressing osteoporosis management gaps, provides an ideal context to investigate moderators’ impact on collaborative learning.

**Objective:**

This study aimed to characterize moderators’ roles and strategies in fostering engagement within health-focused VCoPs, using the Community Fracture Capture Learning Hub as a model.

**Methods:**

A qualitative relational content analysis was conducted on discussion-board interactions across four 6-week cycles (May 2022-October 2023) involving 55 PCPs and 8 moderators. Data included anonymized forum comments of moderators throughout the 4 cycles. Inductive-deductive thematic analysis identified patterns in moderator-participant and moderator-moderator exchanges, focusing on engagement techniques, content facilitation, and interaction dynamics.

**Results:**

Five interconnected themes were identified; (1) participation encouragement: moderators used personalized prompts that highlighted clinical knowledge, affirming language, and balanced encouragement frequency to stimulate involvement; (2) evident moderator collaboration: cross-moderation through mutual collaboration that focused on clinical knowledge, shared prompts, and comment-building reinforced community cohesion; (3) topic discussions: strategic techniques (querying, framing, and real-world contextualization) deepened clinical reasoning; (4) PCPs’ clinical practice discussion: moderators bridged theory-practice gaps using case-based exchanges on screening, treatment protocols, and evidence translation; and (5) progression of engagement: moderators evolved from structured guidance to collaborative dialogue, reducing formality and increasing topic complexity over time.

**Conclusions:**

Thematic analysis suggested that moderators are pivotal in transforming VCoPs into dynamic learning environments. Their ability to blend rational skills, clinical authenticity, and adaptive facilitation drives knowledge coconstruction and practice-relevant engagement. Future studies should consider investing in competency-based moderator training that emphasizes empathetic communication and collaborative techniques as a means of structuring effective VCoPs.

**International Registered Report Identifier (IRRID):**

RR2-10.2196/57511

## Introduction

### Leveraging Virtual Communities of Practice to Foster Interprofessional Education, Knowledge Sharing, and Collaboration Among Primary Care Physicians

Interprofessional education (IPE) is a learning approach in which professionals from different disciplines are educated together to achieve a common goal [1]. As per the World Health Organization, “Interprofessional education (IPE) occurs when two or more professions learn about, from and with each other to enable effective collaboration and improve health outcomes” [2]. Acknowledging the critical role of interprofessional teamwork and collaboration in delivering high-quality patient care, there is increasing advocacy for training health care professionals in collaborative practice skills through IPE, as a supplement to their clinical skill development [3]. IPE has an essential role in breaking down professional silos among primary care physicians (PCPs) [4,5]. Systematic reviews have shown that implementing IPE and interprofessional collaboration (IPC) play vital roles in enhancing the effectiveness of care delivery and contributing to a variety of improved health outcomes [6,7]. One effective strategy for promoting IPE and IPC is the development of communities of practice (CoPs) [8]. These communities bring together individuals with shared interests to work collaboratively, enabling the exchange of information, the transfer of knowledge, the dissemination of best practices, and the strengthening of both professional and interprofessional skills [9]. CoPs are associated with a range of improvements, including immediate advantages such as identification of workforce competencies, knowledge sharing, and reuse of existing knowledge, while also supporting long-term outcomes, including the development of professional identity, increased current awareness, and improved implementation of evidence-based practices [9,10]. With the growth of web-based information-sharing tools, information and communications technology is increasingly being used to promote IPE and IPC, leading to the development of a wide range of virtual communities of practice (VCoPs) [11]. The primary motivation behind VCoPs is to establish networks of individuals who share common interests but are located in different geographic regions [12]. VCoPs have demonstrated effectiveness in several key areas, including the development and implementation of strategies to improve health services and the enhancement of collaboration among PCPs [13,14].

### Enhancing Collaborative Learning in Primary Care: The Evolving Role of Moderators in Virtual Communities of Practice

PCPs encounter considerable difficulties in handling multiple chronic conditions during brief patient consultations, necessitating ongoing learning to maintain the use of evidence-based approaches [15,16]. Consequently, there is an increasing dependence on digital modalities for continuing professional development (CPD) [17-19], as they offer greater flexibility and time efficiency compared with traditional learning methods. Since the COVID-19 pandemic, social media platforms and VCoPs have gained prominence as effective tools for collaborative learning in the health care sector, overcoming time, cost, and geographical barriers [20-22]. VCoPs have been found to reduce professional isolation, enhance workforce retention, promote IPC, and offer a supportive environment that encourages active and safe participation in knowledge exchange [10,12].

Despite their potential, VCoPs often face challenges related to member recruitment, engagement, and long-term sustainability [23]. For VCoPs to be effective, several key factors have been identified as essential, including the presence of external support, strong and supportive leadership, effective facilitation, and the involvement of local champions [9]. Yet, a lack of clarity persists regarding the specific responsibilities of CoP moderators and the appropriate management of power dynamics within CoP groups [24]. We suggest that the role of the moderators in the formation and sustainability of effective VCoPs remains crucial. Evidence suggests that leaders and moderators enhance collaboration by clarifying expectations for participation, maintaining focus within discussions, and fostering an environment characterized by mutual respect, openness, and active engagement [12]. However, a knowledge gap persists in understanding the role of moderators in the success of VCoPs, particularly in relation to the dynamics of their interactions with participants and with one another—an area that remains underexplored in the existing literature. Hence, a full understanding of moderators’ roles needs to be appreciated, so that, as a group, moderators are well prepared and trained, and can provide more strategic approaches that increase participant engagement.

In light of the capacity of VCoPs to serve as a flexible and accessible approach for enhancing PCPs’ knowledge and confidence, and given the limited empirical investigation into the influence of moderators on the effectiveness of VCoPs, this study was guided by the following research question: What roles do moderators play in enhancing the experience of participants in a VCoP?

The objective of the study was to investigate the moderators’ involvement with the program and its members, and identify themes developed in these exchanges.

To answer the research question, in this study, we examined the role of moderators and their contributions in an innovative VCoP known as the Community Fracture Capture (CFC) Learning Hub, which is an interactive, case-based online education platform that facilitates small-group learning through adaptable and customized modules [25]. It is designed to address the osteoporosis treatment gap within community settings, support the adoption of evidence-based bone health practices among PCPs, and enhance the implementation of both the eLearning hub and fracture liaison service models in primary care, both nationally in Australia and internationally [26]. Adopting qualitative analysis of postings and interactions of 8 moderators across 4 cycles (each is 6 weeks long), the study was able to derive a number of emerging themes using qualitative thematic analysis approaches.

## Methods

### Methodology

Full details of the CFC’s development are outlined in our published protocol paper [26].

### Case Study: CFC Hub

The CFC Learning Hub is a secure and adaptable web-based platform designed to support collaborative learning [25,26]. Central to its approach is an interactive Discussion Forum that encourages participant engagement and peer-to-peer exchange on course material. The role of the moderators was specifically important in guiding the discussion. Experienced PCP moderators facilitated the group discussions, with support from specialist moderators as needed, fostering case discussions and directing the conversation based on the content’s relevance and significance. A qualitative evaluation of the CFC model provides important insights into moderators’ interactions with and experiences of this innovative educational approach, enriching quantitative results [27] by offering contextual understanding and a deeper interpretation of the observed outcomes.

Between May 2022 and October 2023, 4 separate 6-week small-group cycles of the program were implemented, involving a total of 55 PCPs. Of these, 33 (60%) PCPs actively participated in the activities by sharing comments or questions. Across all cycles, 8 PCPs and specialist physicians served as the program moderators. At any one time, there was always both a PCP and a specialist active in their respective roles as moderators. All moderators actively contributed to the ongoing discussion through commenting, posting, initiating conversations, or answering enquiries.

Understanding the experiences of the learning activity moderators is crucial for developing and evaluating an effective learning model [28,29]. Effective facilitation of small-group learning is critical for creating opportunities for collaborative engagement among learners and for fostering team-building competencies—skills that are essential for functioning within health care environments [28]. This qualitative study aimed to answer the research question set out above. We propose that moderator engagement within the CFC Learning Hub enhances the platform’s functionality as a peer-driven educational tool by supporting the dissemination of current fracture prevention knowledge to PCPs across a range of professional backgrounds and experience levels, while also contributing to increased confidence and motivation among PCPs in delivering osteoporosis care, with these effects being measurable through integrated analytics systems.

### Ethical Considerations

This study was approved by the Melbourne Health Human Research Ethics Committee (site reference number: 2016.24). All PCP participants provided electronic consent for the use of their anonymized data for research and audit purposes. Electronic informed consent was obtained through Praxhub’s platform, with measures of data anonymization and deidentification in place to uphold privacy and confidentiality standards. Participation in the program was voluntary and did not involve any form of compensation.

### Recruitment

Moderators were recruited by the principal investigators through position announcements circulated within internal networks. Candidates for moderator roles were interviewed informally and engaged in discussions outlining their role responsibilities, alignment with the project’s timelines and milestones, and expectations for engagement. Final selection was based on clinical expertise, prior experience with similar projects, availability, and overall suitability with respect to the project’s aims and team dynamics.

PCP participants were recruited via web promotion of the CFC program via the Praxhub platform [30], joining small private groups (12 to 16 PCPs per cycle) to engage in week-long modules, discussions, and quizzes within a secure, supportive learning environment.

### Participants and Moderators

A total of 55 PCPs participated in the program across four 6-week cycles. Of these, 33 (60%) were active participants, sharing comments or questions. Across all cycles, 8 moderators (2 PCPs and 6 specialist physicians; Table 1) served as the program moderators by leading activities and encouraging discussion. The selected specialists were all clinical endocrinologists with a clinical and research interest in bone and mineral disorders.

**Table 1:** Total number of moderators and their specialty.

CFC^a^ moderators	Number of moderators	Role
Specialists	6	Filter and select relevant case studies. Provide guidance and support clinical focus. Primary focus: content preparation and clinical consultation aligned with up-to-date clinical practice.
PCPs^b^ with special interest in bone health	2	Filter and select relevant case studies. Curate content designed by specialists to ascertain relevance to PCPs. Introduce the topic and lead the group discussion. Primary focus: direct interaction with members to encourage involvement and engagement.

^a^CFC: Community Fracture Capture.

^b^PCP: primary care physician.

### Qualitative Data and Analysis

The qualitative data included in this analysis were collected from over 630 comments, 160 pages, and more than 53,000 words of weekly discussions provided by participants and moderators. In the current analysis, we looked more specifically for moderator-participant and moderator-moderator engagement, with a focus on (1) the nature and tone of moderators’ contributions, (2) the motivation and aims of the moderators’ contributions, (3) interactions between moderators and participants, and (4) interactions between moderators. All the relevant discussion comments were compiled for qualitative analysis.

We used collaborative thematic analysis, as guided by Braun and Clarke [31,32]. The thematic analysis was done by the first and second authors independently initially, after familiarization with the data. This process was carried out to identify patterns in the data. To address the study’s aim, the 2 researchers used relational content analysis [33,34]. Initially, they independently generated codes using both semantic (focused on explicit meanings of the text) and latent (focused on underlying tone and assumptions) approaches to capture a broad range of insights. After initial codes were generated, the 2 authors then collaboratively refined and defined themes and subthemes, through rigorous discussion, based on the evidence from the qualitative data. This process was carried out to identify patterns in the data. In the next phase, these researchers collaboratively reviewed and refined the codes to identify recurring patterns aligned with key themes. These were then organized into subthemes under broader thematic categories. Finally, one researcher coded the full dataset against these subthemes, with a random subset reviewed for accuracy by a senior researcher.

Given the novel approach of the eLearning hub, themes were developed after each cycle and cross-checked against existing and emerging research in the field. As feedback was gathered throughout the program, ongoing refinements were made to both the structure and content of the hub.

A relational content analysis was conducted [33,34], and the findings were organized using both inductive (bottom-up) and deductive (top-down) methods, grouping diverse ideas into meaningful categories. The final themes and subthemes are illustrated with representative quotes from participants.

## Results

### Moderators’ Engagement

**Figure 1:**
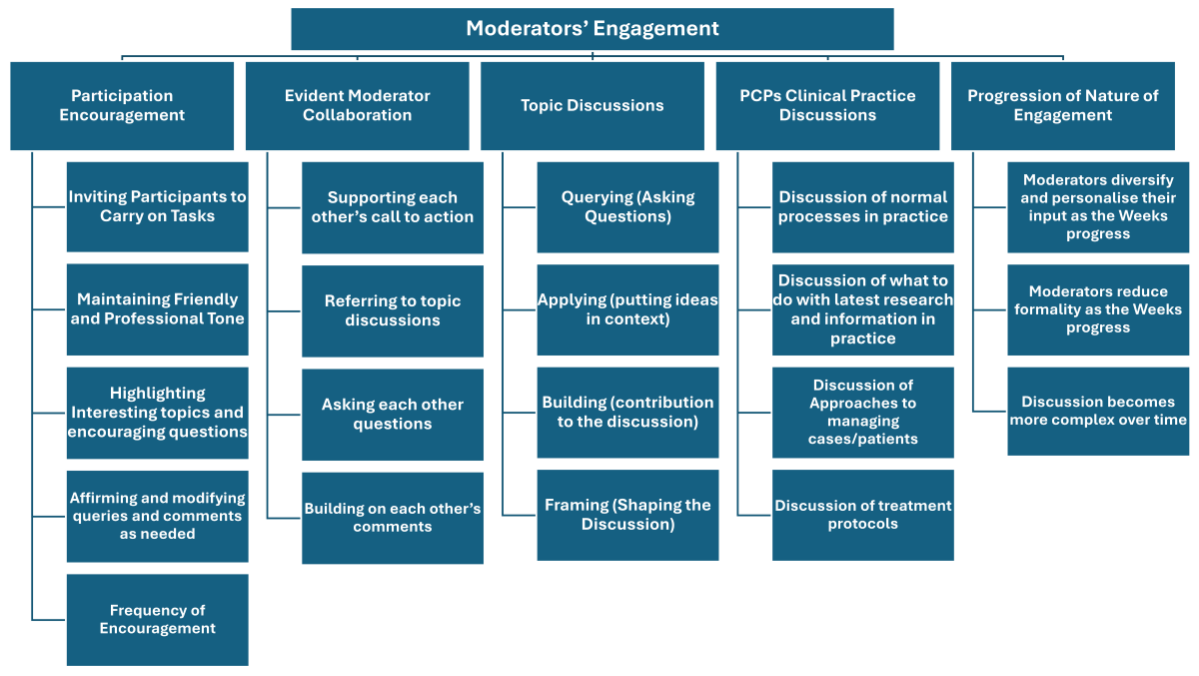
Moderators’ engagement and its main 5 themes and their subthemes. PCP: primary care physician.

This section details the program moderators’ interactions with the program and its members, as well as their engagement techniques. This feedback can be categorized into 5 main themes: participation encouragement, evident moderator collaboration, topic discussions, PCPs’ clinical practice discussions, and progression of nature of engagement (Figure 1).

### Participation Encouragement

#### Overview

Moderators of our virtual learning program used a variety of techniques to foster active participation and engagement among learners. Participants were shown a demonstration of the Praxhub webpage in a virtual, live session to permit interactions with lead moderators and other PCP participants. They actively invited participants to undertake tasks and contribute to discussions, ensuring that the tone remained friendly and professional to create a welcoming environment. By highlighting topics and areas of interest, our moderators aimed to capture learners’ attention and maintain their interest. Encouraging questioning was another key strategy, as it was intended to stimulate continuous engagement with the activity. Additionally, moderators tried to affirm and constructively modify queries and comments, providing clear guidance and support. Striking the right balance in the frequency of encouragement communications was also crucial, aiming to provide learners with timely motivation without feeling overwhelmed. These techniques collectively aimed to help create an interactive and dynamic learning experience. This theme was broken down into 5 subthemes.

#### Inviting Participants to Carry Out Tasks

The moderators applied several key strategies for encouraging participation in the program. Moderators used targeted prompts to guide discussions on specific topics and encourage learners to engage with the material meaningfully. By inviting questions and actively acknowledging past participation, they created a supportive and interactive environment. Other techniques included emphasis on collaborative learning and using prompts for encouraging reflective thinking, further highlighting efforts to foster a sense of community and to stimulate deeper analysis.

One moderator encouraged participation using a targeted prompt:

I would also be very interested to hear your thoughts on strengths and limitations of DXA scans to screen for osteoporosis. If you have other questions, please also feel free to ask them.

Another strategy was to highlight collaborative thinking:

We're really keen to hear your thoughts so that we can all learn from one another.

Also, using prompts for reflective thinking was one of the participation encouragement techniques:

Yes, those are the questions to ask. I'm wondering how others think the answers to those questions might influence what we do next?

Collectively, these approaches aimed to keep learners engaged, motivated, and actively contributing to the learning experience.

#### Maintaining a Friendly and Professional Tone

The moderators’ tone across the activity demonstrated a consistent effort to be friendly, professional, encouraging, and sympathetic. Their informal and upbeat introduction aimed to set a welcoming tone, motivating learners to engage with the interactive hub and reassuring them that participation is confidential. The professional yet warm welcome was targeted at establishing a supportive environment for sharing and learning. Additionally, moderators’ acknowledgment of a participant’s contribution and encouragement for others to submit cases further fostered a collaborative atmosphere. Their affirmations and praise for correct insights and thoughtful responses were intended to be encouraging and supportive, reinforcing positive participation. In addition, their responses, including the appreciation for additional information and structured answers, showed attentiveness and respect for learners’ contributions. They provided personalized responses to participants, highlighting their questions and comments, which was intended to show genuine interest and support, promoting a positive and engaging learning experience.

One moderator gave the following friendly welcome to program participants:

Hi all. The weekend is nearly here. The perfect time to start getting involved in the CFC interactive learning hub. Peer-to-peer learning needs you. And don’t be shy - what happens in the hub, stays in the hub! Ways to get involved?

Another moderator also professionally welcomed participants:

Welcome to the CFC Bone Hub. I'm (name) and I am going to be your course moderator for Week 1. The purpose of this forum is to provide a space where we can learn from each other, through our own experiences, and feel comfortable sharing knowledge in a supportive environment.

One moderator acknowledged a contribution and encouraged others to participate:

Thanks to one of our participants who has provided this good case and some questions to get us all thinking. Don't forget, any participants can send in an interesting case with some questions for consideration to be a case of the week.

Another moderator praised members’ contributions:

You are absolutely right that hyperparathyroidism preferentially results in cortical bone loss.

Also, one moderator expressed appreciation for members’ efforts,

Thanks for that extra information, very helpful in understanding more about the case!

Other moderators personalized responses to different members in a professional and friendly manner:

(Member name)- this is a great point and highlights a crucial component in the case presented.

Overall, the moderators effectively used a blend of encouragement, recognition, and constructive feedback to create a supportive and interactive learning environment.

#### Highlighting Interesting Topics and Encouraging Questions

Moderators tended to highlight and encourage questions to foster participant engagement. They posed questions to introduce new topics and stimulate deeper discussion and exploration. They also highlighted discussing additional topics and cases in an attempt to enrich the discussion with diverse perspectives. Such endorsement of interesting information can enhance the learning experience.

One moderator asked a question to stimulate discussion:

I’d like to ask a question about the fragility fracture risk calculators.

Another moderator used an expression of interest presented by one of the members to highlight the importance of a noteworthy area and to ignite the discussion further:

Hi (member name), Great additional question. I might be pre-empting next week somewhat with a response but since you've asked, I'll give a general overview ahead of the greater detail you'll get in week 6.

A third moderator suggested discussing an additional case vignette alongside the current one to enrich the discussion:

Another complementary case vignette has been submitted for discussion. Perhaps we can discuss it alongside the current case?

Overall, these approaches reflected a strategic effort to engage participants by spotlighting intriguing topics and fostering a dynamic, interactive environment.

#### Affirming and Modifying Queries and Comments as Needed

Moderators worked to affirm and modify queries and comments to ensure clarity and enhance the quality of the discussion. They actively corrected and refined questions or responses to improve accuracy and relevance, ensuring the dialogue remained focused and productive.

For instance, one moderator acknowledged a necessary update to a post by stating, “This is a post edit,” signaling that changes were made for clarity.

Another moderator pointed out a discrepancy in the resources and ensured the correct materials would be provided, saying:

We realised the answers and the resources were not loaded together, so we will be loading these.

To prevent confusion, a third moderator pointed out an error in wording, suggesting a revision:

Note: the following question had an error in the wording which should have read …..

Through these actions, the moderators not only affirmed the ongoing dialogue but also ensured that it was continually refined and improved. Overall, their efforts aimed to help maintain a precise and evolving conversation, fostering an atmosphere of constructive engagement.

#### Frequency of Encouragement

Moderators consistently accessed the discussion boards to monitor and participate in the dialogue, ensuring their engagement at least once daily throughout each cycle. The frequency of their comments and probing questions, aimed at encouraging further discussion, varied based on factors such as the level of response from participants, the depth of the topic being discussed, and the presence of unresolved queries or questions that required follow-up. On average, moderators provided at least one instance of daily engagement per day of the cycle. In addition to their participation in the discussion threads, moderators also sent email communications 3 times a week during the cycle, keeping participants informed about any updates, encouraging them to join in the discussions, and reminding them of the activity’s requirements.

In summary, these efforts contributed to maintaining active participation and fostering a continuous, interactive learning environment throughout the cycle, as demonstrated by the participants’ level of engagement in the activity.

### Evident Moderator Collaboration

#### Overview

The theme of “Evident Moderator Collaboration” covers how moderators worked together to enhance the learning experience through mutual support and active engagement. This teamwork aimed to create a dynamic and cohesive learning environment. This collaboration is subcategorized into 4 subthemes: supporting each other’s call to action, where moderators encouraged participant involvement by backing each other’s prompts; referring to topic discussions, where they recalled earlier posts to create continuity and deepen the conversation; asking each other questions, which modeled inquisitive behavior for participants, encouraging them to engage more deeply; and building on each other’s comments, where they expanded on points made by another moderator or participants to further enrich the dialogue.

#### Supporting Each Other’s Call to Action

Moderators worked collaboratively to encourage participant engagement by supporting each other’s calls to action. They consistently reinforced each other’s prompts, creating a sense of shared responsibility in fostering discussion. This mutual support helped to drive the conversation forward and encouraged participants to respond actively.

For example, one moderator expressed eagerness for participant contributions to emphasize the importance of a colleague moderator’s inquiry and motivated the participants to join the conversation by stating:

Looking forward to hearing your thoughts on (moderator) questions.

In turn, the moderator acknowledged the colleague moderator’s encouragement and shared enthusiasm for productive dialogue by responding:

Thanks (name). I'm looking forward to some good discussion.

As a result, participants were encouraged to continue the conversation and responded effectively to the moderator’s discussion. One of the participants further commented to the moderator, “I like to know how T score to be interpreted with Z score reading.” They then clarified their understanding after receiving the moderator’s response, “Now I understand that if Z score is below -2 then secondary causes should be investigated when the T score is also below -2.5.”

Another moderator also contributed by showing appreciation for another colleague’s efforts and building anticipation for future discussions, saying:

Looking forward to chatting tomorrow night. Thank you (name) for the teriparatide information!

Overall, these interactions highlighted how moderators worked together to strengthen the call for engagement, fostering an atmosphere of cooperation and active participation.

#### Referring to Topic Discussions

Moderators consistently referenced previous conversations to ensure continuity and to build on ongoing discussions. This helped keep the dialogue focused and allowed for deeper exploration of the topics at hand.

As an example, the moderator referred directly to an earlier question regarding the best clinical practice for the management of oligomenorrhoea, demonstrating a clear connection to the ongoing conversation:

With regard to your question about oligomenorrhoea, reduced oestrogen exposure is certainly a risk factor for osteoporosis that should prompt consideration of osteoporosis screening by DXA.

This response addressed a previous inquiry and contributed valuable insight into the current topic, reinforcing the relevance of earlier discussions. By referring to prior exchanges, moderators ensured that the conversation remained cohesive and that each new point was tied back to the broader discussion, enhancing the overall learning experience.

#### Asking Each Other Questions

Moderators demonstrated how inquisitive dialogue can enrich discussions and model active engagement for participants. By posing questions to one another, they not only sought clarification but also encouraged deeper exploration of the topics at hand.

For example, encouraging further discussion and highlighting the value of peer collaboration, one moderator prompted his colleagues by asking:

I wonder (if) (moderator one) or (moderator 2) (can share) what proportion would you think would be expected?

Similarly, a moderator’s question prompted deeper analysis of the case, inviting others to explore the situation more thoroughly, “Great talk (moderator). With the case study - was this a failure? Could we have prevented the fracture? She had low vitamin D, smoking and postmenopausal. Would there be a role for picking her up say 5 years ago and discussing lifestyle, weight training and doing a baseline DEXA if she was willing to pay for it?” which not only sought insight but also invited a peer-driven exchange of strategies.

These exchanges role-modeled the importance of asking questions to clarify, challenge, and deepen understanding, encouraging participants to actively engage in their own inquiries.

#### Building on Each Other’s Comments

Moderators actively expanded on one another’s contributions to deepen the discussion and explore various facets of the topic.

For example, a moderator's comment sparked further engagement from participants:

Great points have been raised in the thread above.

Another moderator responded by acknowledging the discussion and extending it, encouraging the group to look beyond what had already been covered, saying:

Thanks for the comments (moderator one) and (moderator two). We have already discussed some aspects of this case last week, however there are more things to consider

One moderator also built on another moderator’s input and added another layer to the conversation by connecting patient perspectives to the clinical details, saying:

That is so true (moderator name). A lot of my patients associate dietary calcium to high dietary cholesterol.

Overall, through these exchanges, moderators worked together to enhance the depth of the discussion, building on each other’s comments with the aim of leading to more comprehensive and insightful dialogue.

### Topic Discussions

#### Overview

Moderators used a range of techniques to facilitate participant engagement and deepen understanding of the subject matter. Moderators guided participants through the exploration of topics by applying various strategies, which aimed to enhance participants’ comprehension and application of the material. This approach aimed to provide participants with opportunities to actively engage with the content and with one another. This theme is broken down into 4 subthemes.

#### Querying (Asking Questions)

Moderators used questions strategically to engage participants and steer discussions in productive directions. By asking thought-provoking and relevant questions, they encouraged participants to share their knowledge and explore different aspects of the topic. These questions helped highlight key areas for further discussion, often prompting deeper reflection.

For instance, one moderator posed a question to get participants thinking about the management of osteoporosis and setting the stage for a comprehensive discussion, saying:

Can I ask the group for your thoughts about the use of exercise to manage osteoporosis in your patients?

Additionally, one moderator directly engaged a participant and fostered a more interactive environment by asking:

I am surprised by the inclusion of an incident fracture in the first year of treatment as all these medications take time to work and I would have thought a year on any of the treatments would not be long enough to have a real impact? (moderator name), what do you think?

All in all, such questioning techniques effectively stimulated discussion, facilitated knowledge sharing, and ensured that the conversation remained focused and enriching for all involved.

#### Applying (Putting Ideas in Context)

Moderators used real-world examples and practical applications to help participants connect theoretical concepts to their everyday practices. This approach facilitated the discussion by encouraging participants to consider how the topics might play out in their own clinical settings.

For example, one moderator shared, “I also provide my patients with the Healthy Bones Australia fact sheets for calcium and vitamin D...,” using this as an example of how they communicate essential information to patients, which prompted the activity’s moderators and participants to reflect on similar practices.

As a result, one participant contributed by sharing their clinical practice and commented:

Of course, you can start patients … and this is what I do in frail patients…

Additionally, moderators prompted participants to reflect on their observations in clinical practice, as one participant noted:

I always thought that pain from crush fractures was reported much less. It might be the degree of pain! I often see crush # on lateral spines - but no history of severe pain. Women do shrink - again often no specific pain but they all have some pain. What do the docs think about my experience?

This invitation to share personal experiences helped ground the discussion in reality and led to a more nuanced exchange.

Overall, these applications of real-world examples allowed moderators to create a meaningful and relatable discussion, helping participants to apply new ideas and insights directly to their clinical contexts.

#### Building (Contribution to the Discussion)

Moderators focused on building the discussion by encouraging participants to contribute their insights and engage actively with the content. They created an environment where everyone was invited to share, fostering a collaborative atmosphere.

For instance, one moderator used a deliberately leading question to build on an ongoing conversation and encourage participants to consider various implications, asking:

Unfortunately, we don't have any more history on the 1986 fracture but as you say more details would be valuable. A change in therapy is probably warranted but (and it's a deliberately leading question!) are there any potential issues with changing straight from denosumab to teriparatide?

Additionally, moderators prompted deeper engagement by inviting feedback from the group, as seen in the question:

I'm interested if this is consistent with your practice or what you've seen practised? And I'm keen on the group's thoughts as to whether the complexity of the plan is even required.

This approach encouraged participants to reflect on their own practices and share their perspectives, thereby enriching the overall discussion.

By creating these opportunities for input, moderators fostered a sense of ownership and encouraged participants to contribute their experiences and expertise to the conversation, building a more dynamic and collaborative learning environment.

#### Framing (Shaping the Discussion)

Moderators used framing techniques to shape the direction and focus of discussions, ensuring that participants remained engaged and that the conversation stayed aligned with the educational goals. They often introduced topics by setting clear expectations for the flow of the discussion.

For example, one moderator guided the conversation by stating, “Therapies will be covered in depth later in the educational program, but it is worth discussing it briefly for case 2 before we move on to week 3's case,” which helped participants understand the structure and provided context for the current discussion. Another moderator framed the week’s session by acknowledging the contributions of participants, saying:

Welcome to week 3. Thanks to one of our participants who has provided this good case and some questions to get us all thinking. Don't forget any participants can send in an interesting case with some questions for consideration to be a case of the week.

This approach not only shaped the direction of the current week’s discussion but also encouraged ongoing participation from the group.

By encouraging participants to share their thoughts and questions, moderators aimed to create an interactive environment while maintaining focus on key topics. These framing techniques were essential in shaping the overall structure and ensuring that the discussions remained relevant and engaging for all participants.

### PCPs’ Clinical Practice Discussions

#### Overview

Moderators actively shared their own clinical practice experiences to enrich the learning process and make the discussions more relatable. By offering real-world examples from their own practices, they helped bridge the gap between theory and clinical application, which allowed participants to see how they might implement the discussed strategies in their own settings. Sharing such experiences not only helped participants gain a clearer understanding of the clinical aspects of the discussions but also fostered a collaborative learning environment, where participants could learn from each other's real-world experiences. This exchange of clinical knowledge created a richer, more interactive learning experience for all involved. This theme is subcategorized into 4 subthemes.

#### Discussion of Normal Processes in Practice

The moderators shared their normal clinical practices as a way of promoting discussion and encouraging learners to reflect on their own approaches. By presenting real-world scenarios from their practices, they opened the door for valuable exchanges and collaborative learning.

For example, one moderator used a targeted prompt to engage participants with a question on screening and calcium intake, stating:

I will be interested in what people think and how many screen patients before the first fracture. I make it a point to ask all women about calcium intake when coming in for contraceptive advice. I am amazed at how many have insufficient calcium in their diet!

Similarly, another moderator invited discussion around the management of dental procedures in patients treated with antiresorptives:

I’m also keen to hear from colleagues as to how they approach elective dental procedures in patients treated with anti-resorptives. There are at least a few different approaches I’m familiar with in guidelines and from colleagues, am keen to hear from the group on what’s done in the real world.

These strategies encouraged reflection on common clinical practices, fostering a collaborative atmosphere where participants could learn from one another’s experiences and approaches.

#### Discussion of What to Do With the Latest Research and Information in Practice

The moderators facilitated discussions on how to incorporate the latest research and information into clinical practice, encouraging participants to explore new findings and evaluate their practical implications.

One moderator shared insights from recent studies, saying:

That's an interesting question. I'm not familiar with human studies of the effect of ethanol on anti-resorptive actions. This basic science study in rats (study link) found that...,

Additionally, they highlighted other key resources, like the National Prescribing Service Medicine Wise tables, offering practical tools for clinical decision-making:

It is a good resource - for people who have not seen it, it is here: (study link)

Furthermore, one moderator brought in specific research, which led to a deeper conversation about the comparative efficacy of treatments, sharing:

Additionally, one large meta-analysis of 11 studies suggested that while denosumab improves bone density to a greater degree than bisphosphonates in post-menopausal osteoporosis, there is no difference in overall fracture rates

These exchanges helped participants navigate the integration of emerging research into their everyday clinical practice.

#### Discussion of Approaches to Managing Cases/Patients

Moderators encouraged discussion around various approaches to managing complex cases, prompting participants to reflect on their own practices and share insights.

One moderator, for example, shared their surprise at a particularly challenging case, stating:

Wow, interesting case with a very low BMD in the spine - never seen one as low as that! I am interested as to what tests people would do when faced with such a case!

This opened the floor for participants to discuss diagnostic strategies and test selections in similar scenarios.

Another moderator addressed a specific clinical consideration, saying, “For those who manage osteoporosis in patients with renal impairment, you might find the slides in the link useful,” which encouraged participants to explore specialized resources tailored to managing osteoporosis in patients with this condition.

These discussions allowed participants to compare their approaches to managing unique and complex patient cases, fostering a collaborative environment for knowledge-sharing and practical problem-solving.

#### Discussion of Treatment Protocols

The moderators facilitated discussions on treatment protocols, encouraging participants to share their practices and perspectives on various approaches.

One moderator, for instance, initiated a discussion about the approach to managing osteoporosis in postmenopausal women, saying:

Dear all, how many of you organise a DEXA after menopause for women. I find I am performing it a lot more especially if any past risk factors. are there any particular groups that you screen?

This prompted one participant to share real-life clinical experience:

I recently started working in a woman’s clinic. I am struggling to find clear guidance as to when osteoporosis screening should start.

Another moderator contributed by highlighting the relevance of specific guidelines, stating, “The Medicare Benefits Schedule (MBS) guidance on bone densitometry is quite helpful when considering some of the issues raised in this discussion,” inviting participants to consider how these guidelines influence their treatment decisions.

Through these exchanges, participants had the opportunity to gain valuable insights into various approaches, strengthening their collective understanding and enhancing their ability to manage similar cases in their own practices.

### Progression of Nature of Engagement

#### Overview

This theme focused on how the nature of discussions led by moderators evolved throughout the activity. Initially, moderators focused on providing clear instructions and creating a structured environment. Over time, they encouraged more collaborative and reflective dialogue, allowing participants to take on a more active role in shaping the discussions. This theme has been consistently observed throughout the cycles. It is broken down into 3 subthemes.

#### Moderators Diversify and Personalize Their Input as the Weeks Progress

Moderators had to diversify and personalize their input as the weeks progressed, adjusting their engagement to meet the evolving needs of the participants. Early in the activity, the moderators provided general updates and guidance, such as sharing quiz answers and encouraging participants to ask questions. For example, one moderator stated:

Hello everyone, please find the answers to the Week 2 Quiz as below. Please do not hesitate to query the answers, some questions are intended to be controversial! WEEK 2 MCQs (are as follows):

As the activity evolved, the same moderator began tailoring responses to individual participants and their inquiries about the quiz, offering more detailed, personalized feedback, reflecting a shift towards more personalized engagement. For instance, the moderator reshared the MCQs answers directly with one of the participants, saying:

Hi (member name), please see week 2 MCQ answers as below:

And addressing a member’s understanding saying:

Hi (participant name), that is exactly right. Regarding osteopenia paradox: More fractures occur in subjects with osteopenia compared to osteoporosis, because although the relative risk of fracture is higher in osteoporosis, there are more people in the population with osteopenia.

Additionally, affirming a member’s feedback on an answer to a specific question:

Hi (member name), question 9 is designed to be controversial! You are right that anabolic should be considered in all the scenarios below, currently PBS prescriptions require 2 x fragility fractures ( 1 on treatment).

The moderator’s ability to adapt responses based on the participant’s queries reflected progression from general messaging to more personalized, detailed interactions. This evolution in engagement demonstrates a shift towards fostering a more interactive and participant-centered learning environment.

#### Moderators Reduce Formality as the Weeks Progress

As the weeks progressed, moderators gradually reduced the formality in their communication to better engage with participants. In the early weeks, moderators maintained a professional tone, offering structured advice and information.

For instance, one moderator initially provided detailed and formal guidance, saying:

I would still do all the usual screening tests. She could still have other causes for her osteoporosis and could be devastating to miss these.

However, as the activity evolved, the tone became more relaxed and informal, reflecting a shift towards a more conversational and engaging approach. In a later interaction, the same moderator expressed his thoughts in a less formal manner, saying:

OMG I am dizzy just thinking about the case. I would really be looking to the experts for this one... but how is it he is here – he has had renal physicians and endos for years... would they not have intervened early-is it not possible??

This informal tone helped humanize the discussions and made the moderator appear more approachable. By using casual expressions and a less rigid style, moderators fostered a more comfortable environment, encouraging participants to engage more openly and freely in the discussions.

#### Discussion Becomes More Complex Over Time

As the activity progressed, the moderators facilitated discussions that grew increasingly nuanced and complex, challenging participants to consider more detailed aspects of patient care and clinical decision-making.

For instance, a moderator’s questions grew more detailed and reflective over time. At an earlier stage of the cycle, the moderator raised an important, straightforward question about dual-energy X-ray absorptiometry (DXA) screening rates in practice, asking:

Given that all people over age 70 are eligible for a DEXA (DXA), my question to attendees is what proportion of people over 70 in your practice has had a screen?

However, as the cycle progressed, the discussion became deeper, and the moderator’s inquiries became more specific and clinically focused. For example, by mid-cycle, the moderator responded to relevant questions and discussed the routine practice of organizing DXA scans for older adults, pointing out how this had helped in detecting osteoporosis in several cases and highlighting the importance of considering physical signs:

Yes, age over 70 automatically qualifies her. I have been routinely organising DEXAs (DXA) for all my over 70s and have picked a decent amount of osteoporosis. Stooped posture would raise the issue of thoracic crush fractures - a lateral spine in her 60s may have given a diagnosis!

Also, as the discussion went on, the moderator introduced more technical details and deepened the conversation about DXA scans, which led to a more advanced conversation on the variability and interpretation of diagnostic results, saying:

(It is) interesting that the wrist is so different and out of step to the other readings - and Colles fractures are so common and considered pathognomonic of osteoporosis. Most of my DEXAs (DXA) do not include the wrist!

And later in a subsequent cycle, expanding the discussion to more unexplored areas, asking:

For me, one of the issues Is screening around the menopause. I often discuss with women going through menopause getting a baseline DEXA (DXA) scan (self-funded) - particularly to see where they are at. Is that something the rest of you do?

These exchanges demonstrate how the moderators gradually steered the group from general discussions to more complex, case-specific inquiries, encouraging participants to engage with the subtleties of clinical practice and the evolving nature of evidence-based decision-making.

## Discussion

### Principal Findings

Overall, the CFC Learning Hub exemplifies the pivotal role of moderators in cultivating effective VCoPs for PCPs. Our qualitative analysis demonstrated how moderators serve as architects of engagement, bridging theoretical knowledge and clinical practice through strategic facilitation. Data analysis revealed that 5 core engagement themes emerged as pillars of effective moderation: participation encouragement, evident moderator collaboration, topic discussions, PCPs’ clinical practice discussions, and Progression of Nature of Engagement. The study also showed that moderator collaboration was observable through mutual reinforcement of call to action, cross-referencing prior topic discussions, and peer-to-peer questioning and comment-building. In particular, moderators’ engagement evolved in early weeks from formal instructions and structured guidance to informal dialogue, personalized feedback, and participant-led discussions in later weeks. Further, we found that topic discussions were facilitated via strategic querying, clinical contextualization, and progressive framing, where clinical practice discussions dominated exchanges, focusing on screening protocols, interpretation of guidelines, and management of complex cases. These findings align with and extend current understanding of effective VCoP facilitation in health professional education, emphasizing the nuanced interplay between relational warmth, strategic pedagogical techniques, and authentic clinical expertise.

In interpreting our findings through the lens of Wenger’s [35,36] CoP framework, the 5 themes we identified cohere closely with the framework’s theoretical dimensions of mutual engagement, joint enterprise, and shared repertoire. First, participation encouragement and progression of engagement reflect mechanisms by which moderators support mutual engagement: by prompting participation, recognizing contributions, and scaffolding newcomers’ trajectories, moderators help sustain the regular, trust-based interactions that Wenger argues bind a CoP as a social entity (mutual engagement) [37,38]. Second, the themes of topic discussions and Clinical Practice Discussions embody aspects of the joint enterprise, as they signal how community members, under moderator facilitation, negotiate and coconstruct what the CoP is “about”—namely, relevant clinical challenges and professional concerns in practice—thus aligning with Wenger’s notion of a collectively negotiated domain and mutual accountability for its purposes [24,38]. Finally, the evident moderator collaboration theme fosters the development of a shared repertoire, as moderators and participating PCPs jointly build and maintain communal resources (eg, shared norms, language, case-based learning, narratives), which act as artifacts or tools for ongoing practice (eg, stories, tools, and routines) [39]. In sum, our empirical themes suggest that effective moderation not only reinforces Wenger’s core CoP dimensions but also actively operationalizes them, facilitating a more cohesive, participatory, and sustainable community of practice.

The theme participation encouragement demonstrated how moderators proactively created an inclusive environment through targeted invitations, sustained professional yet warm communication, and strategic highlighting of relevant topics. This aligns with evidence that welcoming tones and explicit invitations reduce perceived barriers in web-based learning spaces, particularly for time-constrained professionals [28]. Techniques such as affirming contributions while constructively refining queries echo best practices in digital facilitation, where balancing support with intellectual rigor maintains engagement without compromising depth [40]. Moderators’ daily presence and thrice-weekly email updates further reinforced reliability—a factor linked to sustained participation in web-based CPD [25]. These moderation practices likely satisfy core psychological needs described in self-determination theory (SDT)—autonomy, competence, and relatedness—thereby fostering more intrinsic motivation [41]. Research in online learning shows that perceived instructor (or moderator) support enhances satisfaction of these needs, which in turn promotes engagement [42,43]. By explicitly inviting participants and using clinically relevant framing, moderators support autonomy; by affirming and reflecting, they nurture competence and relatedness. Moreover, their reliable presence establishes trust and predictability, which helps external motivations internalize over time, increasing self-determined engagement. SDT has also been applied in CPD contexts: for example, a study of nursing professionals’ mobile learning found that autonomy and competence were linked to higher motivation in continuing education [44].

VCoP moderators have been shown to enhance collaboration within a VCoP by ensuring clear rules of engagement, maintaining focus in discussions, and fostering engagement, shared respect, and openness [12]. CFC Learning Hub moderators played a pivotal role in sustaining engagement through collaborative questioning and personalized feedback. Their use of relational techniques (eg, affirming contributions or posing reflective questions) mirrors effective practices in web-based facilitation for health professionals [45]. The use of affirming language, reflective prompts, and real-world case discussion aligns with other studies where empathetic communication and clinical relevance were identified as key facilitators of engagement in small-group learning [40]. For instance, their shift from formal instruction to informal dialogue resonates with CoP theory, where trust-building fosters deeper engagement [35,36]. In addition, this shift toward a more informal, trust-based dialogue reflects CoP principles: as trust builds, participants gradually move from peripheral to more central roles (legitimate peripheral participation), deepening their ownership of the community [36,46]. In addition, the use of techniques like framing and query building in topic discussions contributed to fostering activity homogeneity and focus. Reports indicated that a VCoP tends to see higher levels of member interest and involvement when it is more homogeneous or ‘focused’ [47]. Moreover, this facilitation style reflects principles of adult learning theories: adult learners are more engaged when learning is relevant, self-directed, and respectful of their professional experience [48]. By reducing access barriers and signaling respect for participants’ expertise, moderators align with these andragogical principles. Furthermore, moderators’ focus on using real-world clinical examples to contextualize discussions aligns with effective strategies in health-related learning environments [23,47]. Successful VCoPs require that the knowledge shared and discussed by members be applied to clinical practice [47]. Professionals are more likely to engage when the VCoP is patient-centered, and when guidelines and resources for translating research into practice are readily available in user-friendly formats [23].

The evident moderator collaboration theme highlighted how moderators visibly supported each other’s prompts, built upon colleagues’ comments, and modeled inquisitive dialogue. This intentional demonstration of teamwork aligns with Wenger’s [35] concept of “communities of practice,” where shared leadership fosters collective knowledge construction. By publicly engaging in peer questioning, moderators normalized critical thinking and reduced hierarchical barriers, and signaled that inquiry and reflection are communal practices rather than top-down directives. This distributed leadership likely promotes a sense of shared ownership among participants. Moreover, modeling collegial critical thinking may satisfy participants’ relatedness needs, per SDT [41], creating a psychologically safe environment for contribution**.** This finding reinforces the value of cofacilitation in VCoPs, where multiple moderators can diversify expertise and model collaborative problem-solving [10]. Shared moderation builds structural resilience: having multiple moderators reduces reliance on any single individual, which supports long-term sustainability. Over time, as participants observe and internalize this co-leadership, they may increasingly adopt facilitative behaviors themselves, strengthening the community’s capacity and self-governance [40]. Further, the Progression of Nature of Engagement theme illustrated moderators’ evolution from structured guidance to collaborative partnership. Diversifying input (eg, shifting from group announcements to personalized feedback) and reducing formality mirrored community maturation, consistent with VCoP lifecycle models where trust enables deeper interaction [36]. Increasing discussion complexity over weeks reflected adaptive scaffolding—a facilitator skill linked to improved learning outcomes in web-based spaces [28]. Adaptive scaffolding in web-based education was reported to foster learner autonomy, engagement, and self-regulation [49]. From a motivational perspective, this progression supports increasing autonomy (participants gain more control), competence (they engage in complex discourse), and relatedness (peer interactions deepen), all of which facilitate more internalized engagement under SDT. Furthermore, as participants transition from peripheral to more central roles, this reflects the concept of legitimate peripheral participation: newcomers start with less risky participation and gradually assume more central, meaningful roles, reinforcing both individual development and the stability of the community [50].

Our findings underscore that effective VCoP moderation transcends technical platform management; it requires relational, pedagogical, and clinical competencies. Accordingly, training programs for VCoP moderators should emphasize relational skills such as building rapport, maintaining approachable yet professional tones, and fostering psychological safety; pedagogical strategies that include techniques for scaffolding discussions (eg, framing and questioning), encouraging reflection, and modeling collaboration; and clinical authenticity, including the judicious sharing of real-world experiences to contextualize evidence without dominating discourse. Indeed, the presence of a supportive learning environment was reported as a key theme to facilitate the knowledge transfer from CPD to clinical practice [51]. In addition, platform designers should prioritize features that enable seamless moderator collaboration (eg, shared dashboards), personalized communication tools, and analytics to track engagement patterns. A safe, nonjudgmental educational environment; well-organized and structured programs; and the presence of a motivating and intrinsically satisfying atmosphere with smooth and transparent facilitation were among the main factors reported to influence the transfer of knowledge from continuing professional education to clinical practice [52]. The CFC Hub’s success in using case-based discussions also supports designing VCoPs around practice-relevant scenarios rather than abstract topics. These insights additionally carry broader implications for practice, as the identified relational and pedagogical competencies, along with the platform design considerations, can inform moderator training and VCoP implementation across a wide range of clinical specialties and health care settings. For instance, moderation by cognition-based team swift trust was positively correlated with team creativity within nursing education [53]. Developing adaptable training programs and flexible platform architectures may enhance the scalability and relevance of VCoPs, thereby increasing their practical value and supporting more sustainable, cross-disciplinary digital professional development.

### Limitations and Future Research

While this study yields meaningful insights into the role of moderated online learning environments, some limitations should be acknowledged. First, participant-moderator relationships may have been influenced by self-selection bias, with PCPs who were more motivated to improve osteoporosis care likely being overrepresented. Furthermore, participants were recruited via the Praxhub platform, with the majority being metropolitan-based practitioners with over 10 years of clinical experience. This recruitment approach may limit the generalizability of the findings, as the results may not fully capture the program moderation’s impact across more diverse practice settings. Further evaluation is warranted, particularly among regional general practitioners and those with less clinical experience. Additionally, as is common in qualitative research, the study relied on self-reported data, which may be subject to response bias. Future studies should investigate the effectiveness of these educational strategies in larger and more heterogeneous health care populations to better assess their influence on patient care.

Future studies should expand contexts and test these themes in VCoPs across different specialty fields (eg, diabetes and mental health) and geographic settings to identify cross-cutting principles. Future research could also explore how moderator-participant dynamics evolve over extended periods and their impact on community sustainability. Although this study demonstrates immediate engagement and knowledge exchange, it remains uncertain whether these educational gains result in sustained changes in clinical behavior and patient outcomes. Longitudinal investigations are needed to assess the retention of knowledge and skills over time, confidence in providing optimal clinical care in the topics covered, and to evaluate their potential influence on patient management, including relevant clinical outcomes. Further, an in-depth examination of the partnership between the 2 types of moderators is needed, as it would enable a critical analysis of their cross-functional dynamics and offer deeper insight into their complementary roles in delivering an engaging and clinically relevant learning experience.

### Conclusions

This study elucidated the critical role of moderators in VCoPs for PCPs. Through 5 key themes- participation encouragement, evident moderator collaboration, topic discussions, PCPs’ clinical practice discussions, and Progression of Nature of Engagement, we demonstrate how moderators drive engagement by blending relational warmth, strategic facilitation, and clinical authenticity. Moderators’ ability to adapt from structured guidance to collaborative dialogue fosters a dynamic environment where knowledge is coconstructed and applied to real-world practice. These insights strongly suggest the need for dedicated moderator training and thoughtfully designed VCoP platforms that prioritize facilitator support. As digital learning becomes integral to professional development, optimizing moderator roles will be essential for scaling effective, sustainable communities that bridge evidence-practice gaps in health care, strengthen ongoing workforce development, and ensure that VCoPs remain resilient, adaptive, and impactful over the long term.

## Abbreviations

CFC: Community Fracture Capture
CoP: communities of practice
CPD: continuing professional development
DXA: dual-energy X-ray absorptiometry
IPC: interprofessional collaboration
IPE: interprofessional education
PCP: primary care physician
SDT: self-determination theory
VCoP: virtual communities of practice

## Data Availability

All data generated or analyzed during this study are included in this published article and its supplementary information files.
